# Recent Advances in Brain Signal Analysis: Methods and Applications

**DOI:** 10.1155/2016/2742943

**Published:** 2016-09-27

**Authors:** Victor Hugo C. de Albuquerque, Plácido Rogerio Pinheiro, João Paulo Papa, João Manuel R. S. Tavares, Ronaldo Parente de Menezes, Carlos A. S. Oliveira

**Affiliations:** ^1^Programa de Pós-Graduação em Informática Aplicada, Universidade de Fortaleza, Fortaleza, CE, Brazil; ^2^Universidade Estadual Paulista Júlio de Mesquita Filho, Bauru, SP, Brazil; ^3^Instituto de Ciência e Inovação em Engenharia Mecânica e Engenharia Industrial, Departamento de Engenharia Mecânica, Faculdade de Engenharia, Universidade do Porto, Porto, Portugal; ^4^Florida Institute of Technology, Melbourne, FL, USA; ^5^University of Florida, Gainesville, FL, USA

## 1. Introduction

Signal processing and analyses have been extensively used in the field of Neuroscience. For example, (semi)automatic brain-based systems have been increasingly used in various medical applications such as disease prevention, detection and diagnosis of diseases, rehabilitation, smart environments education, serious games and entertainment, security and authentication, biometry, and mobile, as well as new equipment for signal acquisition. These systems are introduced in the literature as accurate, fast, complementary, and alternative devices to aid specialists in their decision making, to facilitate the analysis and interpretation of brain signals, and to reduce and/or eliminate errors [1, 2]. The main objective of this special issue is to promote a discussion on the recent advances related to Brain Signal Analysis from novel methods or applications in order to identify innovative, current, and important contributions to the field of Neuroscience. This special issue of this journal contains 9 original works selected from the 23 submitted. These studies address new trends in novel methods and techniques applied to different applications.

## 2. Computational Intelligence

First of all, I. Martišius and R. Damaševičius developed a prototype of a three-class brain-computer interface system, based on the Steady State Visually Evoked Potentials (SSVEP) paradigm and the Emotiv EPOC headset, to control an online target shooting game implemented in the OpenViBE software. Moreover, S.-K. Kim et al. explored the effects of smartphone push notification delivery during a task according to the level of smartphone overuse, using the event related potential (ERP). The authors concluded that the smartphone presented sensitive reactions associated with notifications during tasks. In addition, J. M. de Oliveira et al. described a virtual environment for patients to engage in a therapeutic game for neuropsychomotor rehabilitation that integrates patient and the proposed virtual environment. In this work, the system recognizes and tracks hands and fingers (Leap Motion sensor) as well as the electroencephalographic sensor (MindWave) responsible for measuring attention levels during task execution. Furthermore, H. G. Yeom et al. examined whether similar rhythmic oscillations with time delays exhibited in macroscopic neural activity at a low frequency during reaching movements from magnetoencephalography (MEG) signals using jPCA showed that the neural mechanism of skilled movements was similar to that of rhythmic movements. Also, K. Yano and T. Suyama presented a novel fixed low-rank spatial filter estimation for brain-computer interface systems applied to recognizing emotions elicited through movies. On the other hand, N. T. Haumann et al. compared results achieved by applying popular methods for reducing artifacts in MEG and EEG signals of the auditory evoked Mismatch Negativity responses in healthy adult subjects. Jointly, R. Grandchamp and A. Delorme proposed the Brainarium, a novel pedagogical and artistic approach based on brain-computer interface technologies, which can deliver and illustrate scientific knowledge, as well as a new framework for scientific exploration. Besides, J. Hori and S. Takasawa proposed an inverse filter that optimizes filtering properties using a sigmoid function applied to human experimental data of visually evoked potentials. These authors concluded that the estimation accuracy is improved and the localized dipole distribution is obtained with less noise. Finally, N. S. Bastos et al. evaluated the use of a data mining technique combined with brain-computer interface systems to assess the behavior of the brain of blind and sighted people in a spatial activity.
